# Transcatheter closure of the patent ductus arteriosus at a public sector hospital in Soweto, South Africa: a review of patient outcomes over 15 years

**DOI:** 10.5830/CVJA-2018-028

**Published:** 2018

**Authors:** Ernest Adams Paul, Cilliers Antoinette, Francis Chersich Matthew

**Affiliations:** Division of Paediatric Cardiology, Department of Paediatrics, Chris Hani Baragwanath Academic Hospital, Soweto, and School of Clinical Medicine, University of the Witwatersrand, Johannesburg, South Africa; Division of Paediatric Cardiology, Department of Paediatrics, Chris Hani Baragwanath Academic Hospital, Soweto, and School of Clinical Medicine, University of the Witwatersrand, Johannesburg, South Africa; Wits Reproductive Health and HIV Institute, Faculty of Health Sciences, University of the Witwatersrand, Johannesburg, South Africa

**Keywords:** patent ductus arteriosus (PDA), intervention, percutaneous, transcatheter

## Abstract

**Background:**

Methods of closing patent ductus arteriosus (PDA) have evolved over time. We review this development in our setting.

**Methods:**

This was a retrospective analysis of children who had transcatheter PDA closure at Chris Hani Baragwanath Hospital between 1993 and 2008.

**Results:**

Over 15 years, 1 254 PDAs were diagnosed, of which 293 required intervention; 139 patients had transcatheter closure, the median age was 1.8 years (interquartile range = 1–4.5 years) and 66% were female (92/139). Mean PDA diameter was 3.2 mm (standard deviation = 1.6 mm), with an average 2:1 shunt. Transcatheter closure was performed using COOKR Flipper coils (n = 93) or Amplatzer™ devices (n = 46). Early occlusion rates for coils were 52% (39/75) and late occlusion occurred in 91% (68/75) of patients. For Amplatzer devices, early occlusion rates were 94% (33/35) and late occlusion was 100%. Amplatzer™ devices, available since 2003, were overwhelmingly used in the later years.

**Conclusion:**

Transcatheter PDA closure was safe and effective in this setting, with outcomes similar to reports elsewhere.

The patent ductus arteriosus (PDA) is a common congenital heart lesion, with isolated PDAs accounting for six to 11% of all congenital heart defects.^1^ Surgical closure of the PDA was the standard management of PDAs for many years,^2^ with the first report of surgical ligation by Gross and Hubbard in 1939.^3^ In the last few decades only, has this been challenged by transcatheter options, which are now the preferred alternatives.^4^,^5^

With the development of new devices, the majority of PDAs can be effectively and safely closed without surgery. This prevents not only complications of the PDA itself, but also the morbidity associated with surgery in general.^1^ The spectrum of PDAs amenable to transcatheter closure continues to increase. With advances in closure technologies, only very small infants with large, symptomatic PDAs, and PDAs with unfavourable anatomy or failed device closure are now candidates for surgical closure.^5^,^6^

The first transcatheter options for closure of the PDA were described by Porstmann and co-workers in 1967.^7^ Since then, the number of available devices has expanded rapidly, most especially in recent years. They range from close copies of the Amplatzer™ design to devices with different shapes and release mechanisms (for example, Ceraflex and Occlutec). The Amplatzer™ devices were first implanted in 1996^8^ and have since been shown to be safe, effective and relatively easy to use.^5^,^9^-^11^ The Amplatzer™ range has increased steadily over time, offering devices with different shapes and even a range to close very small PDAs.^12^

In many settings, the detachable COOKR PDA coils were widely used in small PDAs (< 2.5 mm).^4^,^5^,^8^,^13^ The Amplatzer™ devices, conversely, became increasingly popular for moderate to large PDAs ( > 2.5 mm).^4^,^5^

In this study, we review outcomes of transcatheter closure of PDAs at a large tertiary-level facility in South Africa over a 15-year period, which included the introduction of the Amplatzer™ range of devices. We also document differences between PDAs closed with coils or Amplatzer™ devices, and the shifts in closure methods that occurred over time.

## Methods

A retrospective analysis was conducted of patient records and the Paediatric Cardiology database (Microsoft Access 2003) at the Chris Hani Baragwanath Academic Hospital, Johannesburg, South Africa. All children who had transcatheter or surgical PDA closure at the facility between January 1993 and July 2008 were included in this analysis. A brief update on more recent figures is also provided (January 2009 – August 2017).

After explaining the procedure to the parent or legal guardian, informed consent was obtained. The child was sedated and femoral arterial and venous access was obtained. During the period under study, two groups of transcatheter devices were available, COOKR coils and Amplatzer™ devices. Specifically, the coils used were the detachable MReyeR FlipperR PDA coils, and the Amplatzer™ devices encompassed the Amplatzer™ duct occluders (ADO) and the Amplatzer™ vascular plugs (AVP).

Transcatheter PDA closure was undertaken following standardised procedures, described in full elsewhere.^8^ In brief, a descending aortogram in the lateral position was performed at the start of each catheterisation in order to measure the dimensions of the PDA. This was done to prevent any interference with the PDA that may have caused constriction and inaccurate measurements.

A full diagnostic catheterisation was performed in all patients prior to attempted PDA occlusion; the shunt and pulmonary vascular resistance were calculated. The narrowest point and ampulla were routinely measured and these factors were used to select an appropriate device. With experience, and particularly for Amplatzer™ devices, additional measurements were added. These included the length of duct and diameter of the aorta just proximal to the duct (in smaller children), due to concerns about possible aortic obstruction.

Selection of the size of Amplatzer™ device was based on the manufacturer’s recommendation of at least 2 mm greater than the narrowest point,^14^ but other factors were also considered in device selection, including PDA shape, ampulla size and the size of the aorta. For sizing of the coils, the narrowest point was also taken into account and a coil with a diameter twice the narrowest measurement was chosen. Coils also have a variable number of loops, the number of which depends on the space available in the ampulla of the particular PDA.^15^

Amplatzer™ devices were routinely delivered from the pulmonary side, except in a patient who had an interrupted inferior vena cava, where an Amplatzer™ duct occluder 2 was placed from the aortic side. The majority of coils were delivered from the aortic side; however, if multiple coils were placed in a duct, they were sometimes placed from the pulmonary side, or even using a combination of aortic and pulmonary routes.

Following device placement and prior to release, we performed a number of checks to assess whether the device was correctly placed. A repeat descending aortogram was performed. Pressure gradients in the aorta across the newly closed PDA were measured to ensure there was no significant obstruction to flow in the descending aorta. Once it was ascertained that the device was correctly positioned, it was released. A repeat angiogram was done and a gradient was measured between the ascending and descending aorta to assess any change in position during release that may result in a coarctation. Following the procedure, the patients were observed in the ward until a follow-up echocardiography was done and, provided there were no complications, discharged 24 to 48 hours later.

Patient characteristics consisted of age, gender and anthropometric variables. Clinical details, haemodynamic data, and anatomical details, such as PDA shape and dimensions at echocardiogram and at angiography were used to describe the PDA and resultant haemodynamics. To assess the time taken to close the PDA, the fluoroscopy time for each procedure was captured, where possible. The shape of each PDA was assessed angiographically by reviewing each angiogram and classifying them according to the Krichenko classification (A1-3, B1-3,C1- 3,D,E ).^16^

Failure of transcatheter procedure was defined as any patient requiring a second procedure to effect closure. Early complete occlusion was defined based on an echocardiogram done within 48 hours of the procedure.

## Statistical analysis

Data with a normal distribution were described using means and standard deviations, or medians and interquartile ranges for non-normal data. The χ2 test was used to compare categorical variables, such as patient characteristics and outcomes between those treated with a coil or an Amplatzer™ device. Continuous data were compared using a Student’s t-test (normally distributed data) or Wilcoxon rank sum test (non-normal data).

## Results

Over the 15-year study period, 1 254 PDAs were diagnosed, of which 293 required an intervention to effect closure (23%). Surgical ligation was performed on 167 children and 139 underwent transcatheter closure.

No differences were detected in the demographics of patients who had their PDA closed with an Amplatzer™ device and those who had coiling of their PDA (Table 1). Two-thirds of the study patients were female and the median age was 1.8 years. The anthropometric indices between those who had an Amplatzer™ device or coils to close their PDA were similar. The majority of patients who had a PDA closure weighed more than 6 kg. Amplatzer™ devices were occasionally used in children under 6 kg, even though the product guidelines recommend use only in children above this weight.^14^

**Table 1:** Characteristics of patients who had transcatheter procedures, by closure device

*Variable*	*Total patients (n = 139)*	*Coils (n = 101)*	*Amplatzer™ devices (n = 49)*	*p-value*
Age, median years (IQR)	1.8 (1–4.5)	1.8 (1.0–4.4)	2.1 (1.1–4.5)	0.47
Gender, % (n/N)				
Female	66.2 (92/139)	68.3 (69/101)	63.3 (31/49)	
Male	33.8 (47/139)	31.7 (32/101)	36.7 (18/49)	
Weight, mean kg (SD)	12.8 (7)	13.1 (8.1)	12.9 (9.6)	
< 6 kg, % (n/N)	5.4 (7/130)	5.3 (5/95)	4.4 (2/45)	0.87
≥ 6 kg, % (n/N)	94.6 (123/130)	94.7 (90/95	95.6 (43/45)	0.84
Height, mean cm (SD)	87.4 (22.9)	87.0 (23)	89.2 (24.3)	0.62

N varied due to missing data. The total sum of coils and Amplatzer devices exceeded the number of patients as more than one device was used in some patients. IQR: inter-quartile range. SD: standard deviation.

As shown in Table 2, the average PDA size at its narrowest point was 3.2 mm [standard deviation (SD) = 1.6 mm]. There was a difference in the mean size of coiled PDAs (2.6 mm) compared to those closed with the Amplatzer™ device (4.0 mm). The majority of PDAs under 2.5 mm were closed with coils and the PDAs larger than 2.5 mm were more likely to be closed with the Amplatzer™ device (p = 0.005).

**Table 2:** Characteristics of patent ductus arteriosus and haemodynamic measurements in patients, by closure device

*Variable*	*Total patients (n = 139)*	*Coils (n = 101)*	*Amplatzer™ devices (n = 49)*	*p-value*
PDA size				
Narrowest point, mean (SD)	3.2 (1.6)	2.6 (1.1)	4.0 (1.9)	< 0.001
Size, % (n/N)				0.005
< 2.5 mm	37.5 (42/112)	47.8 (33/69)	21.7 (10/46)	
≥ 2.5 mm	62.5 (70/112)	52.2 (36/69)	78.3 (36/46)	
PDA shape				
A, % (n/N)	72.8 (91/125)	74.1 (63/85)	72.9 (35/48)	0.82
B, % (n/N)	1.6 (2/125)	2.4 (2/85)	2.1 (1/48)	
C, % (n/N)	5.6 (7/125)	3.5 (3/85)	8.3 (4/48)	
D, % (n/N)	2.4 (3/125)	2.4 (2/85)	2.1 (1/48)	
E, % (n/N)	17.6 (22/125)	17.7 (15/85)	14.6 (7/48)	
Chest X-ray				
Cardiomegaly, % (n/N)	85.4 (111/130)	80.2 (73/91)	93.6 (44/47)	0.04
Plethora, % (n/N)	66.2 (86/130)	56.0 (51/91)	83.0 (39/47)	0.002
Haemodynamics				
Pulse pressure, mean mmHg (SD)	46.6 (9.6)	44.9 (10)	48.8 (8.5)	0.03
LA:AO*, mean (SD)	1.6 (0.4)	1.6 (0.4)	1.7 (0.4)	0.5
Shunt, (Qp:Qs)	2.0 (1.2)	1.8 (0.8)	2.5 (1.6)	< 0.001
mean (SD)				
Pulmonary resistance (Woods units), mean (SD)	2.1 (1.8)	2 (1.9)	2.2 (1.6)	0.5
Pulmonary pressure: systemic pressure ratio mean (SD)	0.39 (0.2)	0.4 (0.1)	0.5 (0.2)	< 0.001
Fluoroscopy time				
Median minutes, (IQR)	23.1 (15.3–31.6)	21.3 (14.7–29.8)	23.5 (16.8–32.4)	0.55

N varied due to missing data. The total sum of coils and Amplatzer devices exceeded the number of patients as more than one device was used in some patients. SD: standard deviation; IQR: interquartile range.

Krichenko type A PDAs (conical shaped) dominated, accounting for 73% of cases, with type E (long, tubular with narrowed pulmonary end) the next most frequent at 18%. Type B (short window like) was rare (2% of all patients).

As presented in Table 2, the haemodynamic measurements of the PDAs showed a significant mean left-to-right shunt with a Qp:Qs of 2:1 (SD = 1.2). The mean pulmonary vascular resistance was 2.1 Woods units (SD = 1.8), with a pulmonaryto- systemic pressure ratio of 0.39 (SD = 0.2). Chest X-rays documented cardiomegaly in 85% of the patients, with plethoric lung fields noted on 66% of the X-rays. Several differences were noted in comparisons between haemodynamic features of PDAs closed with coils and with Amplatzer™ devices. Overall, the PDAs where Amplatzer™ devices were used were bigger, with larger shunts and higher pulmonary:systemic pressure ratios.

In about a third of patients, the fluoroscopy time for the procedure had not been recorded. The median fluoroscopy time for those with these data was 23.1 minutes, being similar with coils and Amplazer placement. The shortest procedure took 8.3 minutes and the longest two hours 27 minutes.

Altogether, 150 transcatheter procedures were performed on the 139 patients. More than 80% of patients required only one procedure to effect closure (114), 16% required further intervention (22) and no data on outcomes were available for 2% of cases (three).

Twelve of the 22 patients who required further intervention had a second catheterisation, eight of which were successful and the other four had a residual PDA that was haemodynamically insignificant and it was decided to observe them. A total of eight patients required surgical closure (six with coils and two with Amplazer devices). Two of the 22 were lost to follow up and no further data were available.

**Fig. 1:**
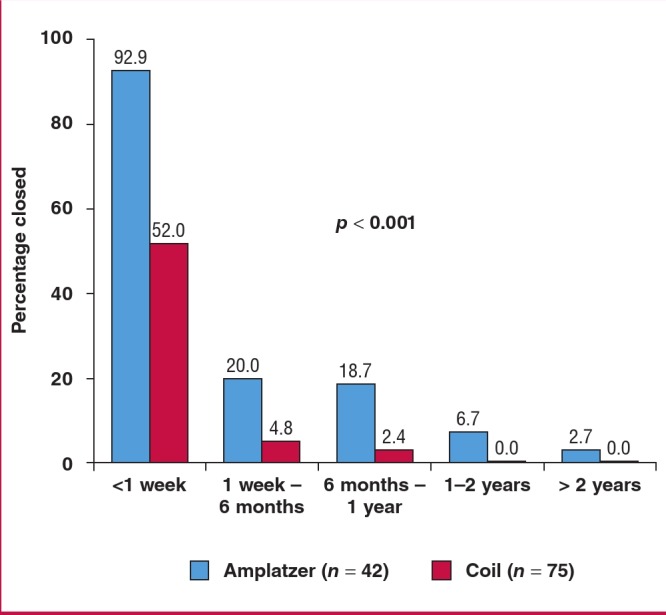
Time to complete closure. Only PDAs in which closure was confirmed on echocardiography were included (p < 0.001 for the comparison between time to closure with Amplazer and coils).

Of the cases where complete PDA closure was documented on echocardiography (117), closure had occurred within one week in over 90% of the Amplatzer™ device cases, but in only half of the procedures using coils (p < 0.001; Fig. 1). Overall, of the whole cohort, no patients had a significant residual PDA in the long run.

**Fig. 2:**
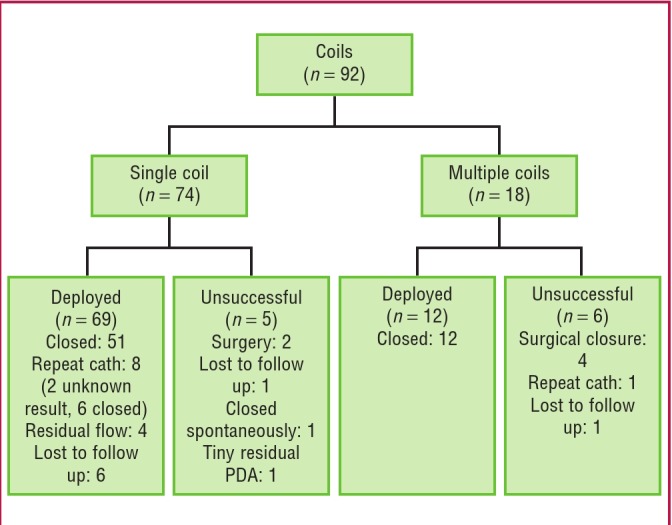
Flow chart describing the patients who had PDA closure with coiling.

A total of 101 COOKR coils were placed in 92 patients who underwent transcatheter PDA closure with a coil (Fig. 2). In the majority of patients, a single coil was used (74), however in larger PDAs (before the Amplatzer™ devices were available) more than one coil was occasionally required. In the 18 patients who required more than one coil at their initial procedure, 13 had two coils, four had three and one had four coils.

Overall, in 78% of patients with a known outcome, the PDA was successfully closed with one or more coils (66/85). Surgical closure was required in six patients where a coil was initially attempted. Five patients had a small residual PDA that did not require surgical closure, one PDA was noted to have closed spontaneously at follow up, and seven patients were lost to follow up.

A total of 49 Amplatzer™ devices were used in 46 patients. Three devices were used during the study period: Amplatzer™ duct occluder 1 (37 patients), Amplatzer™ vascular plug (eight patients), and one patient had an Amplatzer™ duct occluder 2 placed, a device that became available only towards the end of the study period. Closure occurred in 93% of cases where an Amplatzer™ device was used (43/46). In two instances, surgical closure was required and in one patient a tiny residual PDA was present that did not require closure.

Two patients with Amplatzer™ devices required another catheterisation. In the first, an AVP was placed and there was a tiny residual shunt. We attempted to place a coil inside the AVP, which was unsuccessful, but as the residual PDA was very small and insignificant, no further intervention was undertaken. In the other patient, there was a gradient in the ADO pre-release, so the device was removed and the procedure was abandoned. It was later successfully closed with a smaller device.

**Fig. 3:**
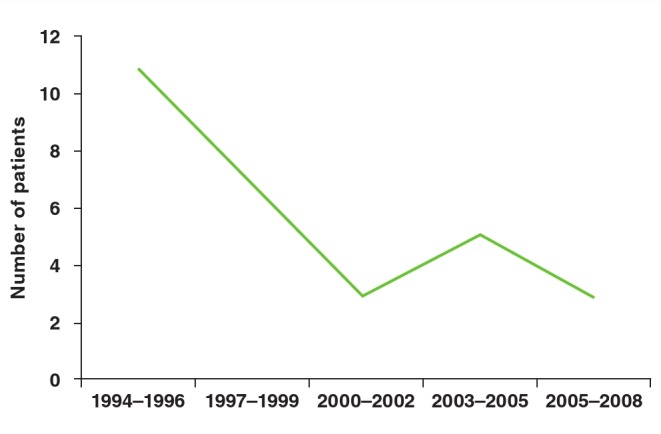
Number of patients requiring a repeat procedure to close the PDA. This graph includes patients who initially had surgery and then went for transcatheter closure.

**Fig. 4:**
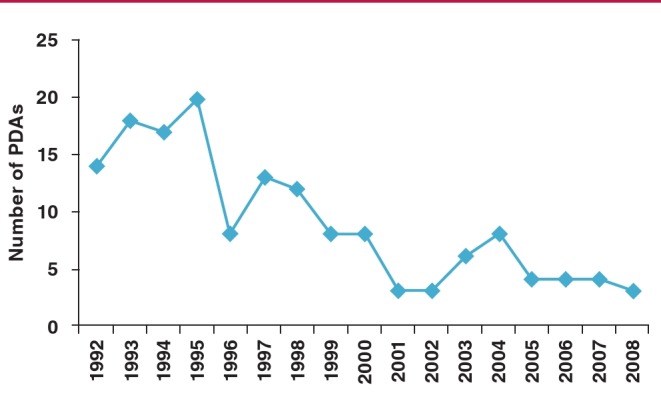
Number of PDAs surgically ligated per year over the study period.

Once Amplatzer™ devices became available at the facility, they rapidly became the preferred device, with the drop in number of coils used mirroring the increase in use of Amplatzer™ devices. The number of patients who had a repeat procedure decreased steadily over the 15 years, except for a brief period where repeat procedures increased around the time the Amplatzer™ device was introduced (Fig. 3). Lastly, the number of patients having their PDA surgically ligated decreased several-fold over the 15-year period (Fig. 4). The age of the child at surgical closure of the PDA also reduced over time, from one year 10 months (1993–1997), to nine months (1998–2002) and then seven months (2003–2008).

Minor complications were noted in 20% of patients (29/145) (Table 3). A total of eight devices embolised (seven coils and one Amplatzer device). None of these resulted in any permanent problems. No differences were detected in the occurrence of either minor or major complications related to the PDA shape.

**Table 3:** Details of minor complications

*Minor complications*	*Number*	*Details*
General		
LV dysfunction	8	6 resolved, 2 lost to follow up
Diminished leg perfusion	2	Resolved with heparin infusion
Arrhythmia	3	SVT – reverted to sinus easily 1 – adenosine 2 – stimulation of RA with catheter
Haemolysis	1	Lost to follow up
Embolisation		
Coils	7	5 coils (4 patients) left in situ – no
	(6 patients)	long-term problems
		2 coils removed by snares
Amplatzer	1	Removed percutaneously
Obstruction and site		
Aorta (n = 2)		
Coil	1	Mild gradient, observed, left in situ
Amplatzer	1	Removed, smaller device used
Pulmonary arteries (n = 6)		
Coil	1	Coil removed
Amplatzer	5	Mild gradient noted, not significant. Devices left in situ
Total	29/145 (20%)	

SVT: supraventricular tachycardia.

Three major complications occurred (2%), all of which resolved. In the first instance, a significant left pulmonary artery obstruction was noted post PDA occlusion. The patient was sent for surgery, the PDA was ligated and the device was removed. The second patient developed complete heart block during the procedure when the balloon on an Arrow–Berman™ catheter burst. Atropine was administered, supraventricular tachycardia followed, which did not respond to adenosine, but following DC cardioversion, sinus rhythm resumed. The PDA was then successfully closed. The third patient had unusual PDA anatomy with a Kommerell diverticulum. The PDA was closed successfully, but on a cardiothoracic angiogram done after the closure, a vascular ring was noted.

## Recent experience with transcatheter closure

From January 2008 to August 2017, a total of 162 procedures were performed at the facility for transcatheter occlusion of PDA. These were predominately done with Amplatzer devices (101 ADO1, 10 ADO2, 14 AVP2 and 6 ADO additional sizes). An additional 22 cases were done with the Occlutech (Occ Duct Occluder devices) and only two with coils. In four cases, the device was removed and replaced during the transcatheter procedure. In total, there were 10 procedures where a device failed. In eight of these cases, the device was removed and the patient then had a surgical procedure, and in the remaining two cases, the device embolised. One was removed in the catheter laboratory, while the other was referred for surgery where the PDA was ligated and the embolised device was removed.

## Discussion

This study shows that transcatheter occlusion of PDAs is safe and effective in our setting. In particular, the introduction of the Amplatzer™ devices raised the safety and effectiveness of closure and a wider spectrum of PDAs can now be closed at the facility. The need for surgical intervention has declined markedly. Reducing the number of these cases by using percutaneous closure of PDAs decreases the time patients with other congenital heart diseases spend waiting for surgery. The data presented for the period 2008– 2017 showed the predominance of Amplatzer devices continued and that few complications occurred with these procedures.

The predominance of females in our study is notable and consistent with what is commonly found in isolated PDAs.^17^ Interestingly, in contrast with Krichenko’s description of PDA shapes, where type B was the second commonest,1 this shape was the least common in our population.

**Fig. 5:**
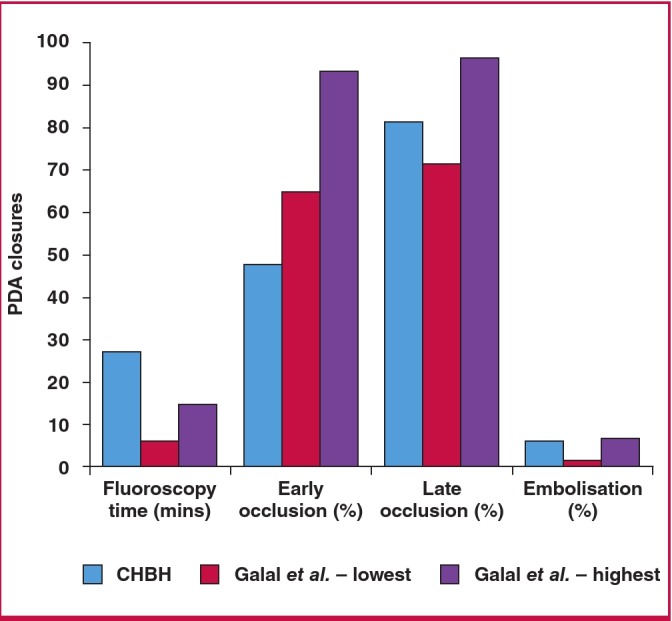
Comparison of PDA closure with coils between our results and the review reported by Galal et al.6 (lowest refers to lowest values in studies reviewed, and highest refers to highest values in studies reviewed).

The findings of our study add to reports from other settings. In 2006, Galal et al. summarised evidence on PDA occlusion from 1995 to 2004, covering 21 articles, which included almost 2 800 patients.6 Our early closure rates are lower than those reported in that review, although definitions of early closure differ between our study and the review (Fig. 5). Late closure rates in our population, however, approximate their reported range. Late occlusion is perhaps the most important outcome measure, as it is a key determinant of whether further procedures are required and of the patient’s risk of endocarditis.^18^ The rate of device embolisation during our study period was acceptable, falling between the highest and lowest rates reported in the review.

In 2004, Pass published a multicentre review of closure of PDAs with the Amplatzer™ duct occluder, covering 484 patients from 25 centres.^19^ Our results are very similar to those of the review, with early closure rates around 90% in the review and in our study. Total closure rates of 97% in our study were almost identical to the 98% in that review. The characteristics of our population and that of the Pass review are similar (e.g. age and weight), as are the PDA sizes and shunt ratios.

While the above studies were done mostly in high-income countries, use of these devices has also been assessed in other middle-income countries.^4^,^5^ A study in Bloemfontein confirmed the effectiveness of coils in 36 patients in a southern African environment.^14^

Notably, our fluoroscopy time was considerably longer than all the results reported by Galal et al.^6^ There are multiple reasons likely account for this finding. Firstly, we perform a full diagnostic catheterisation prior to closing the PDA, while in other settings a briefer catheterisation may have been done, focused on only closing the duct. Secondly, the introduction of a new device (Amplatzer) necessitated a considerable learning curve for the catheterisation team, prolonging the procedure for the initial patients. The slight increase in number of patients requiring a repeat procedure to close a PDA around the time that the Amplatzer devices were introduced was also likely due to the learning curve.

Finally, our facility is a nationally accredited paediatric cardiac training centre, with a high turnover of trainees who each need to perform several procedures in order to become proficient, and to complete their sub-speciality. As a result, these procedures most often involve close supervision and teaching of trainees with little or no experience. The long fluoroscopy time is an important finding, and results in increased radiation exposure to both patients and staff. Clearly, attention needs to be paid to strategies to reduce radiation exposure in our setting.

## Study limitations

Types of procedures and outcomes of current care may differ from that of our study, given that our data collection ended in 2008 and new devices have been introduced since then. Nevertheless, these data describe the important transitions in our unit from one type of device, coils, to the Amplatzer range, and from surgery to transcatheter closure. Furthermore, this study is limited in that it is a retrospective record review. While efforts were made to capture all available data, some information was missing. Similarly, most patients who underwent surgery prior to the era of device closure did not undergo cardiac catheterisation so the data relating to PDA size and shape were not available for these patients, limiting our ability to compare these and other patients.

## Conclusions

Transcatheter closure of PDAs in the catheterisation laboratory at Chris Hani Baragwanath Academic Hospital is both safe and effective. The treatment outcomes are similar to those in highincome countries, which have considerably more resources for treating their patient populations. The introduction of a new device, although associated with a learning curve, broadened the range of PDAs that could be closed and improved closure rates. Efforts are needed to address the factors influencing fluoroscopy times. As more devices become available, the range of PDAs that could be closed will likely further increase.
